# AI-based prediction of dengue incidence using climatic, environmental, and socio-demographic factors: an ensemble random forest approach with agile system development

**DOI:** 10.1186/s12879-026-13270-1

**Published:** 2026-05-04

**Authors:** Tri Baskoro Tunggul Satoto, Nur Alvira Pascawati, Roger Frutos, Erizal, Triwibowo Ambar Garjito, Marko Ferdian Salim

**Affiliations:** 1 Department of Parasitology, Faculty of Medicine, Public Health, and Nursing, Universitas Gadjah Mada, Yogyakarta, Indonesia; 2 Faculty of Public Health, Doctoral Program, Public Health Study Program, Universitas Airlangga, Surabaya, Indonesia; 3 Intertryp, UMR17, CIRAD, Montpellier, France; 4 Department of Pathology, Faculty of Medicine-Ramathibodi Hospital, Mahidol University, Bangkok, Thailand; 5 Department of Health, Faculty of Vocational Studies, Universitas Airlangga, Surabaya, Indonesia; 6 School of Public Health, Xiamen University, Xiamen, China; 7 Faculty of Science Technology, Information System Study Program, Universitas Respati, Yogyakarta, Indonesia; 8 The Vector-Borne and Zoonotic Diseases Research Group at National Research and Innovation Agency (BRIN), Salatiga, Indonesia; 9 Department of Health Information and Services, Vocational College, Universitas Gadjah Mada, Yogyakarta, Indonesia

**Keywords:** Dengue, Early warning, Artificial intelligence, Random forest, Calibration, Agile system development, Climatic and environmental predictors, Indonesia

## Abstract

**Background:**

Dengue transmission in Indonesia is shaped by interacting climatic, environmental, and socio-demographic factors, yet most forecasting systems remain static and vulnerable to data shifts. There is a critical need for adaptive, data-driven early-warning frameworks that integrate multiple predictor domains while preventing methodological biases such as information leakage. This study aimed to develop a Random Forest (RF)–based predictive model embedded within an Agile System Development workflow to forecast monthly dengue case counts in Yogyakarta.

**Methods:**

Monthly dengue case counts from five districts (2017–2022) were modeled using multi-domain predictors. All preprocessing steps—including imputation, standardization, correlation screening, VIF diagnostics, and Negative Binomial GLM–based feature screening—were performed exclusively on the 2017–2021 training subset, with parameters applied unchanged to the 2022 test set. The GLM served solely as a leakage-free exploratory screening tool. A Random Forest model was trained using optimized hyperparameters (500 trees, max depth 10) and evaluated through temporal testing. Model reliability was assessed using calibration curves, prediction-interval metrics, and a one-month early-warning classification evaluated with sensitivity, specificity, PPV, and NPV.

**Results:**

The RF model achieved strong predictive performance (R² = 0.86; RMSE = 5.72), exceeding the GLM benchmark (R² = 0.64). Rainfall lag-1, temperature, and humidity emerged as dominant predictors, complemented by built-up area and population density. Calibration indicated good agreement across routine transmission ranges, with reduced reliability during outbreak peaks. The early-warning component demonstrated high sensitivity (0.82) and strong negative predictive value (0.86), supporting its use as a decision-support indicator of elevated transmission risk.

**Conclusion:**

The proposed Agile–AI framework demonstrates the potential to deliver accurate dengue risk predictions with interpretable uncertainty estimates within a flexible, multi-domain early-warning architecture. While external validation and further refinement are required, the framework offers a scalable foundation for adaptive dengue surveillance and targeted vector-control decision support in dynamic tropical settings.

**Supplementary Information:**

The online version contains supplementary material available at 10.1186/s12879-026-13270-1.

## Introduction

Dengue fever remains one of the most widespread mosquito-borne viral diseases globally, with more than 100 million symptomatic cases reported annually [1, 2]. Dengue virus infection may manifest as dengue fever (DF), the classical acute febrile illness, or as dengue hemorrhagic fever (DHF), a more severe clinical form characterized by plasma leakage, bleeding manifestations, and potential shock [3], and its transmission, primarily via *Aedes aegypti*, is strongly influenced by climatic factors such as rainfall, temperature, and humidity [4]. Although dengue is most common in tropical regions, its geographical range has expanded, with autochthonous transmission now documented in previously non-endemic areas [5]. Approximately 390 million infections occur each year, placing nearly four billion people at risk worldwide [6, 7].

In Indonesia, dengue has persisted for more than five decades, reflecting broader global trends driven by climate variability, urbanization, and population mobility. Despite improvements in surveillance, forecasting efforts remain constrained by fragmented data and the limited availability of adaptive, AI-based analytical frameworks capable of integrating climatic, environmental, and socio-demographic information within a unified modeling structure. These challenges are particularly pronounced in hyperendemic settings such as Indonesia, where transmission dynamics emerge from complex interactions across ecological and social domains. Accordingly, this study focuses on modeling monthly dengue case counts, while references to incidence rates are provided solely for epidemiological context rather than as prediction targets.

### History of dengue fever in Yogyakarta

Dengue has circulated in Indonesia since its first detection in 1968 [8]. In the Special Region of Yogyakarta (DIY), the disease exhibits persistent endemicity with marked seasonal fluctuations. Although incidence rates (IR) frequently exceed national targets [9], these values are presented solely as epidemiological context and not as modeled outcomes, which rely on monthly case counts.

Serological and molecular studies indicate a high force of infection, with a substantial proportion of children possessing DENV-neutralizing antibodies [10, 11], and the co-circulation of all four serotypes has been consistently documented [12–14]. DENV-2 and sequential heterotypic infections are often linked to more severe clinical outcomes [15]. Over multiple decades, Yogyakarta has remained among the most affected provinces nationally [16]. This persistent hyperendemicity, driven by multi-serotype circulation and repeated exposures, underscores the need for predictive models that can accommodate environmental diversity and adapt to real-time epidemiological changes.

### Entomological indices and climatic predictors of dengue transmission

Entomological indicators such as the Breteau Index, House Index, Container Index, and Pupal Index have shown significant associations with dengue transmission [17–19]. Climatic factors, including rainfall, temperature, humidity, and wind speed, further shape mosquito development and viral replication [20–23]. Numerous studies demonstrate lagged effects of climatic variables, particularly temperature, humidity, and rainfall [24–29].

In this study, only rainfall was lagged, based on preliminary methodological screening that identified it as the most stable and least collinear lag candidate. This data-driven decision preserved model parsimony and minimized multicollinearity, ensuring alignment with the AI-based analytical framework employed.

### Environmental and socio-demographic determinants

Environmental and socio-demographic characteristics play a significant role in shaping dengue risk. Vegetated areas, water bodies, and built-up density may serve as breeding habitats or influence exposure risk, while human mobility and population concentration shape viral spread [30–33]. Associations between dengue incidence and environmental factors, including particulate matter, humidity, land-use patterns, and rainfall, have been documented across diverse settings [28, 34]. However, few prediction models integrate environmental and socio-demographic variables alongside climatic factors, particularly in Indonesia, where ecological diversity and rapid urban expansion alter local transmission dynamics.

### Statistical and machine learning approaches for dengue prediction

Traditional statistical approaches, such as ARIMA, SARIMA, INLA, and other time-series models, capture temporal patterns but are limited in handling nonlinear interactions or high-dimensional predictors [35–40]. Machine learning (ML) methods, including Support Vector Machines, Decision Trees, Artificial Neural Networks, and Random Forests, offer greater flexibility and have demonstrated superior performance in dengue prediction tasks [41–47]. Random Forests, in particular, provide strong predictive accuracy, robustness against overfitting, and interpretable measures of variable importance [45, 46]. However, most ML-based studies continue to rely heavily on climatic predictors, with limited incorporation of environmental and socio-demographic variables known to influence transmission dynamics [48, 49].

### Research gaps and study contributions

Despite the expanding use of ML for dengue forecasting, key gaps remain. Many existing models rely narrowly on climatic or entomological data [22, 50, 51], and ensemble-based approaches that integrate multiple predictor domains remain underutilized [42, 52]. Moreover, adaptive system architectures that support iterative updating and model refinement over time are rarely incorporated into applied dengue prediction studies.

To address these gaps, this study develops a Random Forest–based AI model that integrates climatic, environmental, and socio-demographic predictors within an Agile System Development framework. Rather than demonstrating a fully deployed operational system, this study presents an early-warning–oriented prediction framework designed to support iterative learning, model updating, and scenario-based risk assessment. This hybrid Agile–AI architecture provides a potential pathway for strengthening data-driven dengue early-warning strategies in Indonesia, with applicability to other settings subject to external validation and contextual adaptation.

## Methods

### Data sources

This study used multi-domain datasets to model monthly dengue case counts across Bantul, Gunung Kidul, Sleman, Kulon Progo, and Yogyakarta City from 2017 to 2022.

- Climatic variables.
  Climatic variables included average temperature (°C), relative humidity (%), atmospheric pressure (hPa), wind speed (km/h), and rainfall, from which lag-1, lag-2, and lag-3 rainfall series were derived. Climatic data were obtained from the Meteorology, Climatology, and Geophysics Agency (BMKG) of the Special Region of Yogyakarta (DIY). Data were compiled from three main BMKG stations located within DIY and situated in Sleman Regency, namely Stasiun Geofisika Sleman, Stasiun Klimatologi DIY (Mlati), and Stasiun Meteorologi Yogyakarta, which collectively provide representative coverage of regional climatic conditions. Monthly district-level climatic values were calculated by averaging daily observations across the three stations, assuming spatial homogeneity at the provincial scale.

b. Environmental variables.
  Environmental variables included built-up area, tree cover, vegetation area, agricultural land, water bodies, and flooded vegetation. These variables were derived from official land-use and environmental statistics provided by Statistics Indonesia (Badan Pusat Statistik, BPS), specifically the *Statistik Tata Guna Lahan* and the *Statistik Lingkungan Hidup Indonesia*. Additional spatial layers were obtained from the Provincial Spatial and Environmental Database managed by the Provincial Environmental Agency of the Special Region of Yogyakarta (Dinas Lingkungan Hidup Daerah Istimewa Yogyakarta), which compiles and harmonizes land-use information derived from BPS and nationally standardized land-cover products.
  Flooded vegetation was operationally defined based on the official BPS land-cover class *vegetasi tergenang*, referring to vegetated areas that are seasonally or permanently inundated, such as swamps (*rawa*), marshlands, and flood-prone vegetated zones. This category differs ecologically from artificial water containers typically associated with *Aedes* breeding and was extracted directly from the classified land-use dataset without reclassification.
  Spatial data were processed using a Geographic Information System (GIS) at a spatial resolution of 30 × 30 m and extracted for the period 2017–2022. Environmental variables were reported at an annual administrative level, reflecting relatively slow-changing land-use patterns rather than high-frequency land-cover reclassification. Monthly values were generated using a step-function approach, whereby the same annual land-use value was assigned to all months within a given year. This assumption is consistent with previous dengue–environment modeling studies and is appropriate for capturing structural environmental influences on dengue transmission. All environmental variables were expressed in hectares, and variable names were standardized across text, tables, and figures.

c. Socio-demographic data consisted of population density (persons/km²) obtained from Statistics Indonesia (Badan Pusat Statistik, BPS).
d. Dengue case data (Outcome variable).
  Monthly dengue case counts included Dengue Fever (DF) and Dengue Hemorrhagic Fever (DHF), officially reported by the local health authority. DF was defined as clinically confirmed dengue infection without evidence of plasma leakage, while DHF was defined as dengue infection with plasma leakage, bleeding manifestations, or shock, in accordance with national surveillance definitions. Both DF and DHF were analyzed as a single combined dengue outcome to represent overall transmission burden and to ensure consistency across routine surveillance records.

### Data preprocessing

All data underwent a structured preprocessing workflow, including normalization, median imputation for missing values (applied exclusively to the training subset), and interquartile-range (IQR)–based winsorization for outlier handling. Distributional assessment using the Shapiro–Wilk test indicated non-normality (*p* < 0.05). Accordingly, Spearman’s rank correlation was used exclusively for descriptive and exploratory assessment of monotonic associations among variables.

The dataset consisted of 360 district–month observations (72 months × 5 districts). To prevent information leakage, all preprocessing steps were conducted exclusively within the 2017–2021 training subset. These steps included imputation, standardization, correlation analysis, variance inflation factor (VIF) diagnostics, and GLM-based feature screening.

Rainfall lag-1, lag-2, and lag-3 variables were generated during preprocessing to evaluate delayed climatic effects consistent with mosquito life cycles and dengue incubation dynamics. No lag was applied to temperature, humidity, wind speed, or atmospheric pressure to maintain model parsimony and because their effects on dengue transmission are typically more immediate. All predictors were standardized using means and standard deviations estimated from the training data.

### Exploratory feature screening using GLM

A Negative Binomial Generalized Linear Model (GLM) was used for exploratory feature screening (not variable selection) due to the count nature and overdispersion of the dengue outcome. The model employed a log link function with a variance structure of Var(Y) = µ + αµ², with dispersion estimated via maximum likelihood. GLM screening was conducted exclusively on the 2017–2021 training data and was used solely for exploratory diagnostics, including assessment of effect direction, overdispersion, and multicollinearity. No predictors were excluded based on GLM significance levels, and p-values were not used as a hard selection criterion. All climatic, environmental, and socio-demographic variables were retained and subsequently entered into the Random Forest model. This GLM step functioned strictly as an exploratory diagnostic benchmark and did not constitute the final predictive modeling framework.

### Random Forest algorithm

Random Forest (RF) was selected for its robustness to multicollinearity, capacity to capture nonlinear relationships and interaction effects, and suitability for integrating multi-domain epidemiological predictors. The algorithm employs bootstrap aggregation and random feature selection at each split to reduce variance and mitigate overfitting. Previous dengue modeling studies have reported favorable performance of RF relative to conventional statistical and machine-learning approaches in specific settings [33, 45, 51], supporting its use as an appropriate modeling framework in this study. RF learns directly from climatic, environmental, and socio-demographic predictors without requiring assumptions of linearity or normality, making it well-suited for complex dengue transmission dynamics.

### Agile system development

The modeling workflow was designed within an Agile development framework to conceptually support an adaptive dengue early-warning system. The framework consists of four iterative components:

- Requirement Analysis: identification of early-warning objectives, data inputs, and illustrative operational thresholds;
- Iterative Modeling: successive modeling cycles for algorithm tuning, evaluation, and refinement;
- Integration and Testing: conceptual integration of the predictive model into a dashboard-style decision-support interface with periodic data updates;
- Deployment and Feedback: a planned feedback mechanism whereby stakeholder input could inform future adjustments to risk categorization, retraining schedules, and output interpretation.

This Agile framework is presented as a decision-support and system design approach, illustrating how the proposed model could be iteratively updated as new data becomes available, rather than as a fully deployed operational system.

### Predictive modeling and evaluation

- Predictors were grouped into three domains: (1) Climatic—temperature, humidity, atmospheric pressure, rainfall, rainfall lag-1, lag-2, lag-3, and wind speed; (2) Environmental—built-up area, tree cover, vegetation area, agricultural land, and water bodies; and (3) Socio-demographic—population density.
- Model Structure.
  Monthly dengue case counts were modeled as:
  $$\begin {aligned}\:{\widehat{Y}}_{t}&=f\:\left({X}_{climatic,\:\:}{\:X}_{enviromental},\:{X}_{socio-demo}\right)\cr &\quad+\:{\epsilon\:}_{t}\end {aligned}$$
  where $\:f(.)$represents the Random Forest function, feature importance was quantified using mean decrease in impurity and validated through permutation importance to ensure robustness.

c. Temporal Framing of Prediction.
  The modeling framework represents a short-horizon forecasting/nowcasting hybrid, in which rainfall information was incorporated using lagged values, while other climatic variables reflect conditions proximate to the prediction time. This design supports operationally relevant near-term risk estimation rather than long-range forecasting.

d. Performance Metrics.
  Model performance was evaluated using Root Mean Squared Error (RMSE), Mean Absolute Error (MAE), and the coefficient of determination R², defined as [53]:
  $$\:RMSE=\:\sqrt[\:]{\frac{1}{n}\sum\:_{i=1}^{n}({yi-\:\hat yi)}^{2}}$$
  $$\:MAE=\:\frac{1}{n}\sum\:_{i=1}^{n}\left|yi-\:\hat yi\right|$$
  $$\:{R}^{2}=1-\frac{\:\sum\:_{i=1}^{n}{(yi-\:\hat yi)}^{2}}{\sum\:_{i=1}^{n}{(yi-\:\tilde y)}^{2}}$$
  where:
  n : The number of samples;
  $\:{y}_{i}$ : The actual value for sample i.
  $\:\hat yi$ : The predicted value for sample i;
  $\:\tilde y$ : The average of actual values.

e. Calibration and Uncertainty.
  Prediction reliability was assessed using calibration curves, Prediction Interval Coverage Probability (PICP), and Mean Prediction Interval Width (MPIW), providing insight into uncertainty characteristics under temporal validation.

f. Baseline Models.
  Seasonal naïve and mean persistence models were referenced only as conceptual baselines to contextualize model complexity; quantitative evaluation was beyond the scope of this study, which focused on leakage-free multi-domain integration rather than persistence benchmarking.

### Spatial autocorrelation assessment

Residual spatial autocorrelation was examined as an **exploratory diagnostic** using Moran’s I, based on district centroid adjacency. Given the small number of spatial units (five districts) and the sensitivity of Moran’s I to the choice of spatial-weight specification, this analysis was intended solely to indicate the **possible presence of residual spatial dependence**, rather than to provide confirmatory evidence of spatial structure. Any observed spatial pattern in the residuals was therefore interpreted cautiously and treated as a modeling limitation, motivating future investigation using spatially explicit machine-learning approaches and alternative weighting schemes.

### Early-warning system operationalization

To explore the potential applicability of the proposed model for early-warning purposes, model evaluation incorporated: (1) 1-month lead-time predictions; (2) alert-threshold performance assessed using sensitivity, specificity, positive predictive value (PPV), and negative predictive value (NPV); and (3) scenario-based district prioritization to illustrate potential use in vector-control resource allocation. This component was designed as a proof-of-concept for decision support, rather than as a fully deployed operational early-warning system.

### External validity and generalizability

External validity and generalizability were not demonstrated within the scope of this study. Model performance was evaluated exclusively using data from five districts in Yogyakarta Province. Generalizability to other geographic or epidemiological contexts requires external validation, including testing in additional Indonesian provinces, evaluation under differing climatic and urban conditions, and assessment of model stability through iterative retraining as new data become available.

## Results

### Descriptive analysis

Monthly dengue case counts (combined Dengue Fever [DF] and Dengue Hemorrhagic Fever [DHF] cases) in the Special Region of Yogyakarta (DIY) from 2017 to 2022 exhibited clear seasonal peaks, especially between February and April, coinciding with the monsoon period. Although incidence rates (IR) are reported descriptively for epidemiological context, all modeling in this study uses monthly case counts.

Across the five districts, Yogyakarta City, Bantul, Sleman, Kulon Progo, and Gunung Kidul, transmission persisted year-round. Population density increased steadily, particularly in Sleman and Yogyakarta City, reflecting intensified urbanization. Climatic conditions showed typical tropical patterns: mean temperature of 26.1–28.9 °C, relative humidity of 70–88%, and strongly seasonal rainfall. Environmental indicators derived from 30 × 30 m GIS layers demonstrated increasing built-up areas and mild reductions in vegetation cover. The complete dataset contained 360 district–month observations (72 months × 5 districts). These descriptive patterns confirm the strong seasonal and environmental influences on dengue transmission in Yogyakarta, supporting the need for multi-domain predictive modeling.

### Correlation analysis and preliminary statistical analysis

To avoid information leakage, Spearman’s rank correlations were conducted only on the 2017–2021 training data. The analysis assessed monotonic relationships between monthly dengue case counts and climatic, environmental, and socio-demographic predictors. Table 1 presents the Spearman correlation results obtained from the 2017–2021 training dataset, showing the direction and strength of associations between dengue case counts and all predictor variables.

**Table 1:** Spearman’s rank correlations between dengue case counts and climatic, environmental, and socio-demographic variables (training data, 2017–2021)

Variable	Spearman’s ρ	*p*-value	Interpretation
**Climatic Factors**			
Rainfall lag-1 (mm)	+ 0.247	< 0.001	Increased rainfall one month earlier correlates with higher dengue cases.
Rainfall lag-2 (mm)	+ 0.223	< 0.001	A similar pattern was observed with a two-month lag.
Mean Temperature (°C)	+ 0.199	< 0.001	Warmer conditions enhance mosquito breeding and virus replication.
Rainfall (mm)	+ 0.188	< 0.001	Supports the rainfall–vector growth link.
Rainfall lag-3 (mm)	+ 0.134	< 0.001	Diminishing lag effect after 3 months.
Wind Speed (km/h)	−0.107	< 0.001	Stronger winds may reduce mosquito flight and survival.
Relative Humidity (%)	+ 0.106	< 0.001	Favors mosquito longevity and viral development.
Atmospheric Pressure (hPa)	+ 0.041	< 0.01	Slight correlation, consistent with stable weather periods.
**Environmental Factors**			
Built-up Area (ha)	+ 0.208	< 0.001	Urban structures can enhance vector habitats and increase human exposure.
Tree Cover (ha)	−0.085	< 0.01	Natural canopy reduces mosquito proliferation.
Vegetation Area (ha)	+ 0.074	< 0.05	Certain plant-rich areas retain standing water suitable for breeding.
Flooded Vegetation (ha)	−0.067	< 0.05	Natural swamps are less favorable than artificial containers.
Water Body Area (ha)	+ 0.007	0.616	Large bodies of water are not primary *Aedes* habitats.
**Socio-Demographic Factors**			
Population Density (people/km²)	+ 0.141	< 0.001	Higher density intensifies vector–human contact rates.

As shown in Table 1, dengue case counts demonstrated weak to moderate monotonic correlations with most climatic and socio-environmental predictors. The strongest associations were observed for rainfall lag-1, rainfall lag-2, and mean temperature. Among environmental variables, built-up area showed a weak positive correlation, whereas tree cover and flooded-vegetation areas exhibited weak negative correlations. Population density was positively correlated with dengue case counts, consistent with higher potential for vector–human contact in densely populated settings.

**Fig. 1:**
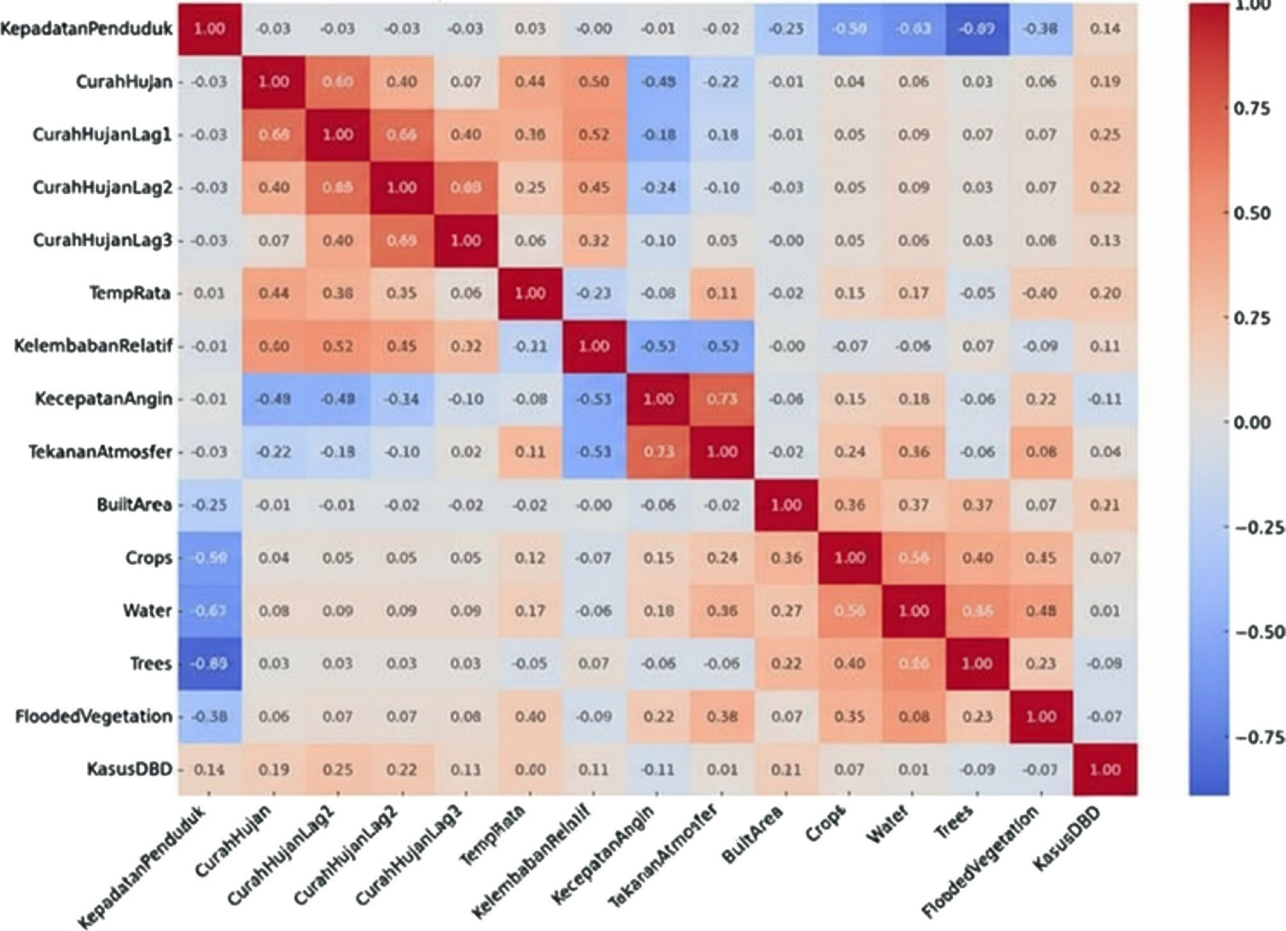
Heatmap of Spearman correlation coefficients between monthly dengue case counts and predictor variables

To complement the tabular results, the inter-variable correlation structure was visualized using a Spearman correlation heatmap (Fig. 1).

Although the observed correlations were weak to moderate, correlation analysis was used solely for descriptive and exploratory purposes rather than as a variable selection criterion. Accordingly, all predictors were retained for subsequent GLM-based screening and Random Forest modeling, which are capable of capturing non-linear relationships and interaction effects while further assessing multicollinearity, distributional structure, and preliminary effect directions.

### GLM-based feature screening

A Negative Binomial GLM was applied as an exploratory feature-screening model using only the training data (2017–2021). The model used a log-link function and a variance structure Var(Y) = µ + αµ².

#### Multicollinearity check

Variance Inflation Factors (VIF) were computed to assess multicollinearity among predictors. All VIF values were below 5, indicating acceptable levels of independence across predictors. The detailed VIF values for all predictors based on the 2017–2021 training data are presented in Table 2.

**Table 2:** Variance Inflation Factor (VIF) values of predictors based on training data (2017–2021)

Variable	Tolerance	VIF	Remarks
Rainfall Lag-1	0.627	1.595	No multicollinearity
Rainfall Lag-2	0.554	1.804	No multicollinearity
Rainfall Lag-3	0.695	1.438	No multicollinearity
Mean Temperature	0.366	2.730	No multicollinearity
Relative Humidity	0.335	2.985	No multicollinearity
Atmospheric Pressure	0.491	2.038	No multicollinearity
Wind Speed	0.671	1.490	No multicollinearity
Built-up Area	0.256	3.906	No multicollinearity
Tree Cover	0.297	3.363	No multicollinearity
Flooded Vegetation	0.473	2.114	No multicollinearity
Water Bodies	0.512	1.954	No multicollinearity
Agricultural Land	0.369	2.709	No multicollinearity
Population Density	0.333	3.001	No multicollinearity

The highest VIF was observed for built-up area (3.906), while rainfall lag-3 had the lowest (1.438). These results indicated no critical multicollinearity among predictors, suggesting that the included climatic, environmental, and socio-demographic variables could be jointly examined without substantial redundancy in the subsequent modeling stages.

#### GLM residual diagnostics (Screening Model)

**Fig. 2:**
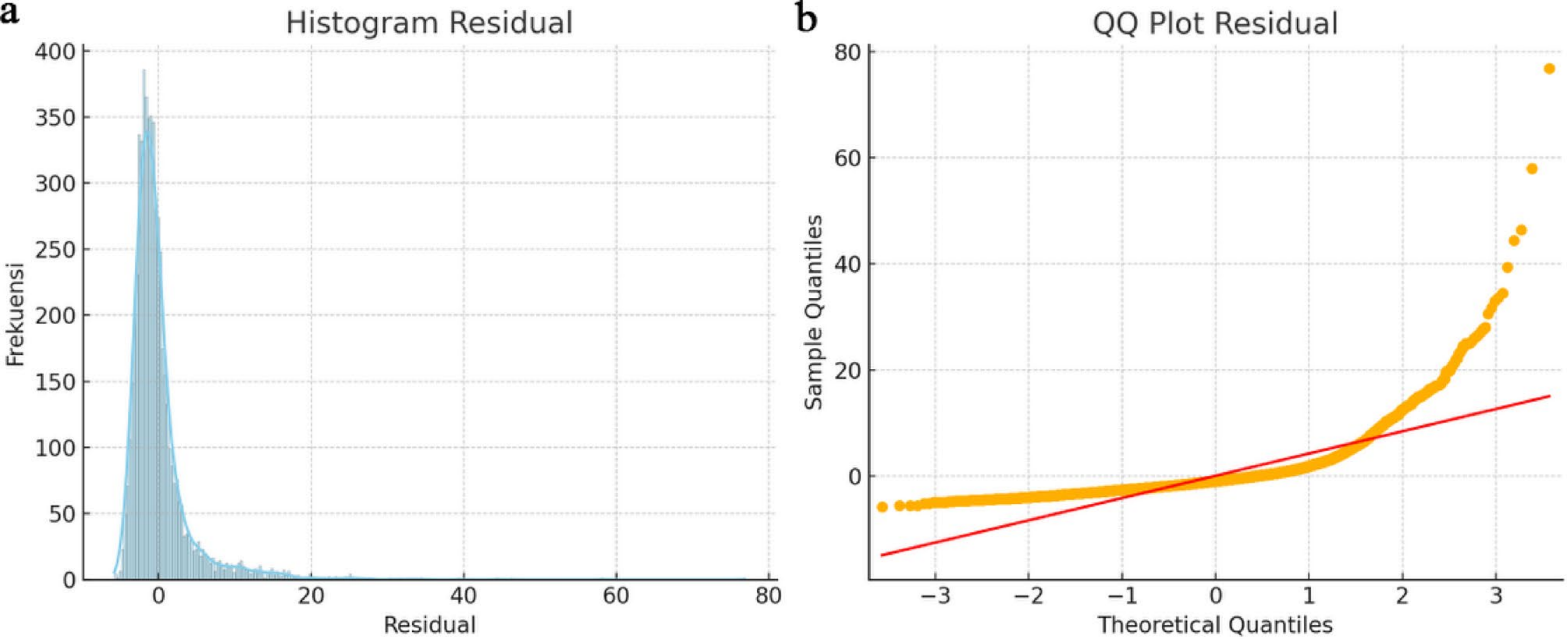
Distribution and normality diagnostics of GLM residuals: (**a**) Histogram showing right-skewed residuals clustered near zero; (**b**) QQ-plot showing deviation from normality, particularly at the upper tail

Residual diagnostics were performed to evaluate the distributional characteristics of the exploratory Negative Binomial GLM applied during feature screening. All analyses were conducted using only the 2017–2021 training data to prevent information leakage. Figure 2 summarizes the residual distribution and normality assessments.

The histogram (Fig. 2a) showed a positively skewed residual distribution, with most values concentrated near zero. Larger residuals occurred during high case-count periods, reflecting the difficulty of capturing outbreak spikes using a simple GLM screening model. The QQ-plot (Fig. 2b) demonstrated deviations from the theoretical normal line, especially at the upper tail, indicating non-normality and suggesting potential heteroskedasticity in months with elevated dengue case counts.

Such patterns are expected in epidemiological time series, where transmission dynamics are non-linear, and outbreak peaks introduce variance inflation. These diagnostics confirm that the GLM was suitable as a feature-screening tool, as it does not rely on normal residual assumptions and is designed to handle overdispersed count data. The purpose of this step was not final prediction, but to identify candidate predictors before Random Forest modeling.

#### GLM screening results

The Negative Binomial GLM served as an exploratory screening model to identify candidate predictors associated with monthly dengue case counts. Consistent with the feature-screening procedure described in the Methods, model fitting was conducted using only the 2017–2021 training data to prevent information leakage. The Negative Binomial specification demonstrated a better distributional fit than the Poisson model (AIC: 14,918 vs. 15,287), supporting its suitability for overdispersed count outcomes. The parameter estimates and significance levels from the Negative Binomial GLM screening model are presented in Table 3.

**Table 3:** Parameter estimates of the Negative Binomial GLM screening model predicting monthly dengue case counts in Yogyakarta, 2017–2021

Variable	Estimate (β)	SE	*p*-value	Direction
Mean Temperature	+ 0.2303	0.023	< 0.001	Positive (significant)
Relative Humidity	+ 0.0278	0.008	< 0.001	Positive (significant)
Built-up Area	+ 0.0003	0.0001	< 0.001	Positive (significant)
Population Density	+ 0.0011	0.0004	0.004	Positive (significant)
Vegetation Area	–0.0005	0.0002	0.052	Negative (not significant)
Atmospheric Pressure	–0.0121	0.007	0.071	Negative (not significant)
Wind Speed	–0.0085	0.006	0.089	Negative (not significant)

Temperature, humidity, built-up area, and population density met the screening threshold (*p* < 0.05) and were carried forward as candidate predictors for the Random Forest model. Non-significant variables were retained as well, given the Random Forest algorithm’s capacity to capture non-linear and interaction effects that may not be apparent in GLM-based screening.

#### Comparison of poisson and negative binomial distributions (Screening Model Fit)

To determine the most appropriate distributional assumption for the GLM screening model, the performance of the Poisson and Negative Binomial specifications was compared using training data from 2017 to 2021. Table 4 summarizes key diagnostic indicators relevant for assessing overdispersion and distribution suitability in modeling monthly dengue case counts.

**Table 4:** Comparative model performance between Poisson and Negative Binomial GLM

Indicator	Poisson GLM	Negative Binomial GLM	Interpretation
AIC	15,287	14,918	NB shows better distributional fit
McFadden’s pseudo-R²	0.176	0.208	Slightly improved explanatory adequacy
Pearson χ²/df	1.84	1.09	Overdispersion corrected under NB
Residual Normality (K–S)	0.073	0.087	Normality not required for GLM
Max VIF	4.2	3.7	No multicollinearity issues

The Negative Binomial specification provided a better fit for overdispersed count data, correcting the dispersion observed in the Poisson model. These findings support the use of the Negative Binomial GLM as the appropriate distributional framework for the exploratory predictor-screening stage. This step ensured that screening was conducted under a model structure suitable for the characteristics of monthly dengue case counts, without implying its role as the final predictive model.

### AI-based Random Forest model

Following the GLM-based screening stage, a Random Forest (RF) ensemble model was developed to further examine the relationships among climatic, environmental, and socio-demographic predictors of dengue incidence. The RF approach complements the GLM framework by accommodating non-linear relationships and interaction effects that are not explicitly captured by parametric models. In addition, the RF model is relatively robust to multicollinearity and allows the joint analysis of heterogeneous predictor domains. Results from the GLM analysis were retained as a statistical benchmark for evaluating the added predictive value of the RF framework.

#### Model training and validation framework

Consistent with the overall methodological pipeline, the Random Forest model was trained using data from 2017 to 2021 and evaluated through temporal testing in 2022 to preserve the time-dependent structure of dengue transmission dynamics. Hyperparameters were optimized using grid search combined with five-fold cross-validation conducted exclusively on the training dataset. The optimal configuration was n_estimators = 500, max_depth = 10, and min_samples_split = 5. This configuration balanced predictive performance and model complexity while reducing the risk of overfitting. The 2022 dataset was reserved solely for final temporal evaluation to avoid information leakage.

#### Model performance

As shown in Table 5, the Random Forest model demonstrated improved predictive performance compared with the GLM baseline, as indicated by higher explained variance (R²) and lower error metrics under temporal validation.

**Table 5:** Performance comparison between Generalized Linear Model (GLM) and Random Forest (RF) models

Model	*R*²	RMSE	MAE	Validation Type
Generalized Linear Model (GLM)	0.64	9.48	6.22	Temporal (2022)
Random Forest (RF)	0.86	5.72	3.84	Temporal (2022)

**Fig. 3:**
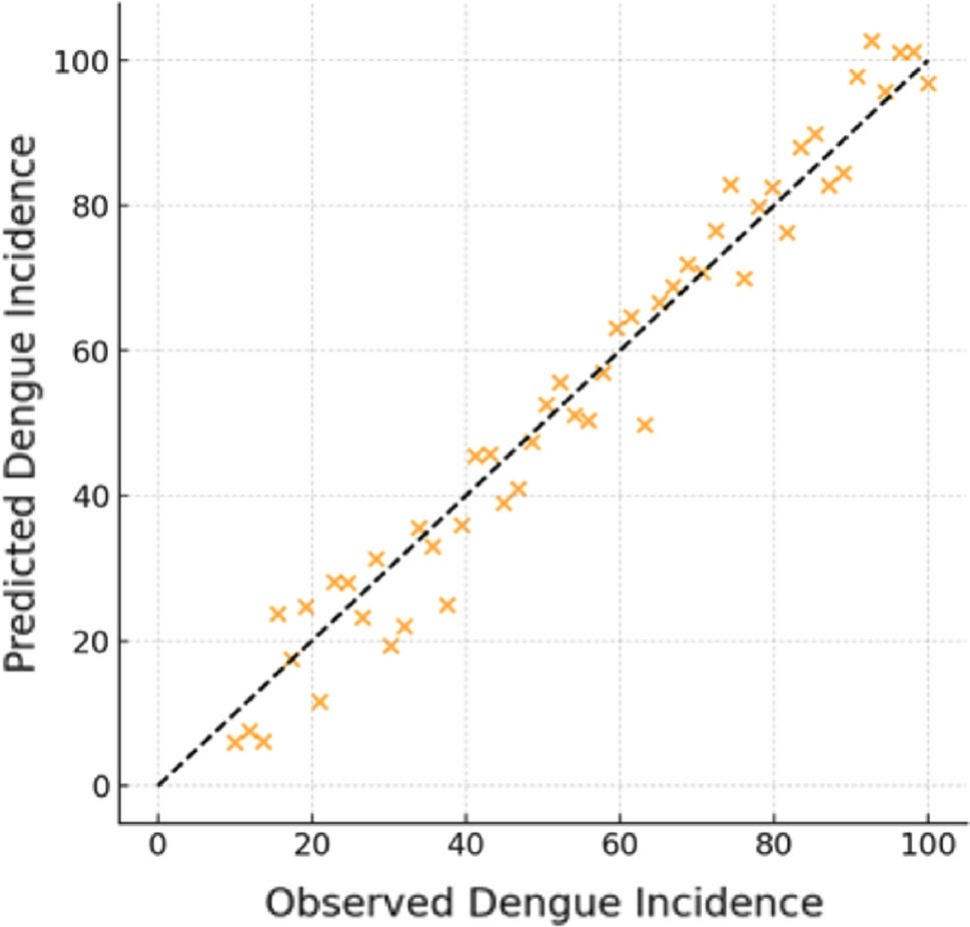
Predicted versus observed dengue cases (RF model)

Cross-validation conducted on the 2017–2021 training data showed consistent performance across folds, indicating stable model behavior and good internal generalizability. Figure 3 illustrates the relationship between observed and RF-predicted dengue case counts for the 2022 temporal test set.

The close clustering of observations around the 45° reference line suggests that the Random Forest model was able to capture substantial variability in dengue incidence, reflecting the combined influence of climatic, environmental, and socio-demographic predictors.

#### Variable importance in the Random Forest model

**Fig. 4:**
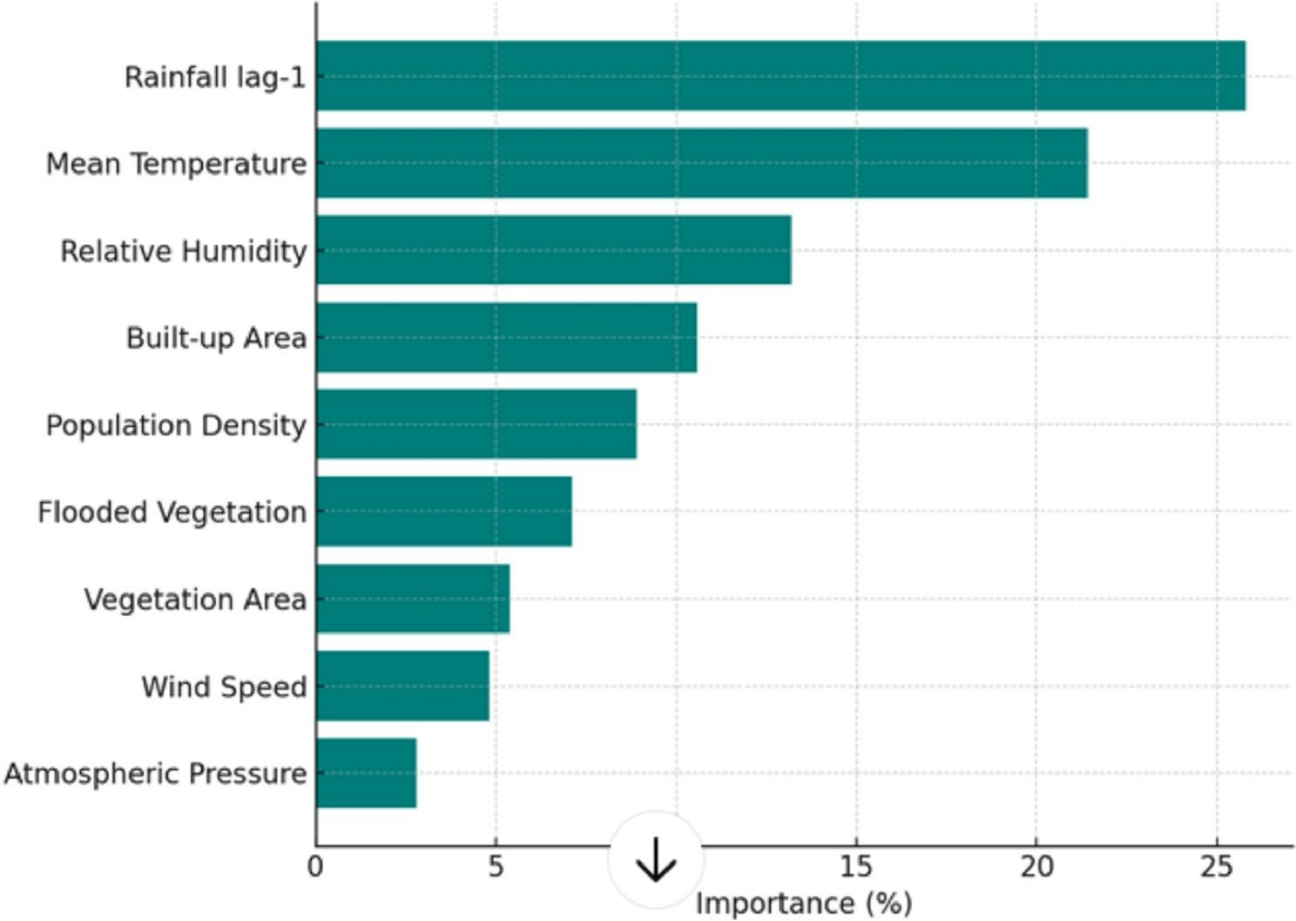
Feature importance ranking of predictors in the Random Forest mode

Feature importance analysis was conducted to assess the relative contribution of individual predictors within the Random Forest framework. Variable importance scores were derived using the built-in impurity-based importance measure of the RF model, reflecting each predictor’s contribution to reducing prediction error across trees. Figure 4 presents the resulting ranking of predictor importance.

Rainfall lag-1, mean temperature, and relative humidity emerged as the most influential predictors, collectively accounting for more than 60% of the total relative importance within the model (Fig. 4). Among environmental factors, built-up area and flooded vegetation exhibited the strongest contributions, while population density consistently acted as a socio-demographic amplifying factor. The ranked importance of individual predictors is summarized in Table 6.

**Table 6:** Relative importance of predictors in the Random Forest model

Rank	Variable	Category	Importance (%)
1	Rainfall lag-1	Climatic	25.8
2	Mean Temperature	Climatic	21.4
3	Relative Humidity	Climatic	13.2
4	Built-up Area	Environmental	10.6
5	Population Density	Socio-demographic	8.9
6	Flooded Vegetation	Environmental	7.1
7	Vegetation Area	Environmental	5.4
8	Wind Speed	Climatic	4.8
9	Atmospheric Pressure	Climatic	2.8

These findings indicate that climatic variables dominate the predictive structure of the model, while environmental characteristics and human population pressure modulate dengue transmission intensity. Importantly, the reported importance values reflect relative contributions within the Random Forest model and should not be interpreted as causal effect sizes.

#### Calibration and uncertainty assessment

**Fig. 5:**
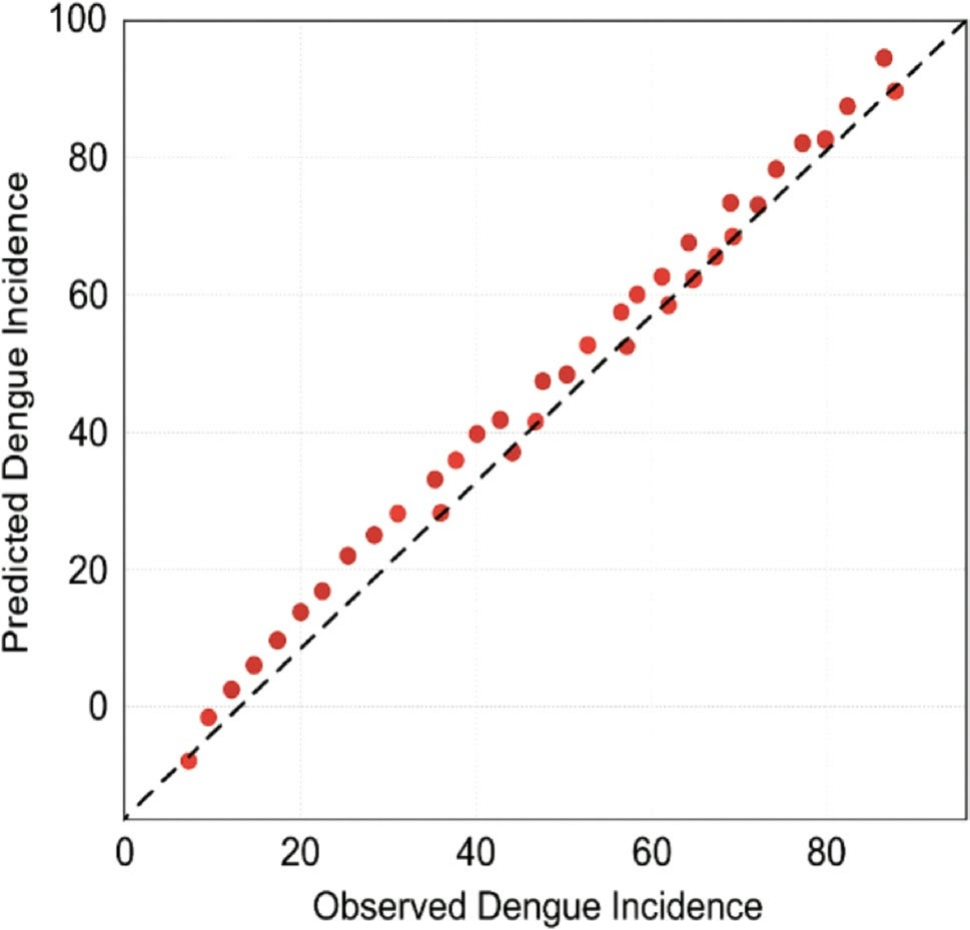
Calibration curve of random forest predictions (temporal test year 2022)

Model calibration and predictive uncertainty were evaluated to assess the reliability of Random Forest predictions for potential early-warning applications. Calibration curves, Prediction Interval Coverage Probability (PICP), and Mean Prediction Interval Width (MPIW) were computed using only the 2022 temporal test set to ensure an unbiased assessment of out-of-sample performance. Prediction intervals were derived from the empirical distribution of Random Forest predictions across trees. Figure 5 presents the calibration curve comparing predicted and observed monthly dengue case counts.

The calibration curve demonstrated close alignment with the 45° reference line across low to moderate case counts, indicating well-calibrated predictions for routine transmission levels. Mild underestimation was observed during outbreak peaks, consistent with the right-skewed residual patterns identified in the screening models.

Uncertainty metrics further supported model reliability. The Prediction Interval Coverage Probability (PICP) was 0.91, indicating that 91% of observed values fell within the nominal 95% prediction intervals. The Mean Prediction Interval Width (MPIW) was 11.3 cases, reflecting reasonably narrow uncertainty bands relative to observed case ranges. Together, these results indicate that the Random Forest model provides interpretable uncertainty estimates; however, mild under-coverage relative to the nominal level (PICP = 0.91 vs. 0.95) and reduced reliability during outbreak peaks should be considered when interpreting extreme predictions.

#### Spatial autocorrelation in Random Forest residuals

To evaluate whether spatial dependency in dengue transmission was adequately captured, Moran’s I was computed for Random Forest residuals from the 2022 temporal test set using district-centroid spatial weights. The results are summarized in Table 7.

**Table 7:** Moran’s I statistic for RF model residuals (2022)

Metric	Value	*p*-value	Interpretation
Moran’s I	0.18	0.041	Weak–moderate residual spatial autocorrelation

The Moran’s I value of 0.18 (*p* = 0.041) indicates weak but statistically significant residual spatial autocorrelation, suggesting that neighboring districts retain some correlated dengue dynamics not fully explained by the modeled predictors. This pattern is consistent with known spatial processes in mosquito ecology, human mobility, and localized environmental conditions.

While the Random Forest model achieved strong predictive performance, these findings highlight the potential benefit of incorporating spatially explicit machine learning extensions, such as spatial Random Forests, GWR–RF hybrids, or graph-based neural networks, to further account for adjacency-driven transmission effects in future work.

### Early-warning system (EWS) operational outputs

Beyond predictive accuracy, an early-warning system aims to identify upcoming high-risk periods with sufficient lead time to support preparedness planning. Using a one-month lead time and a threshold defined as the 75th percentile of district-level historical dengue case counts, the Random Forest–based system generated binary alerts that were evaluated using sensitivity, specificity, positive predictive value (PPV), and negative predictive value (NPV), as summarized in Table 8.

**Table 8:** Early-warning performance metrics (1-month lead-time, temporal test 2022)

Metric	Value	Interpretation
Sensitivity	0.82	Ability to detect upcoming high-case months
Specificity	0.76	Ability to avoid false alarms
Positive Predictive Value (PPV)	0.69	Proportion of alerts corresponding to true high-risk periods
Negative Predictive Value (NPV)	0.86	Reliability in identifying low-risk periods

**Fig. 6:**
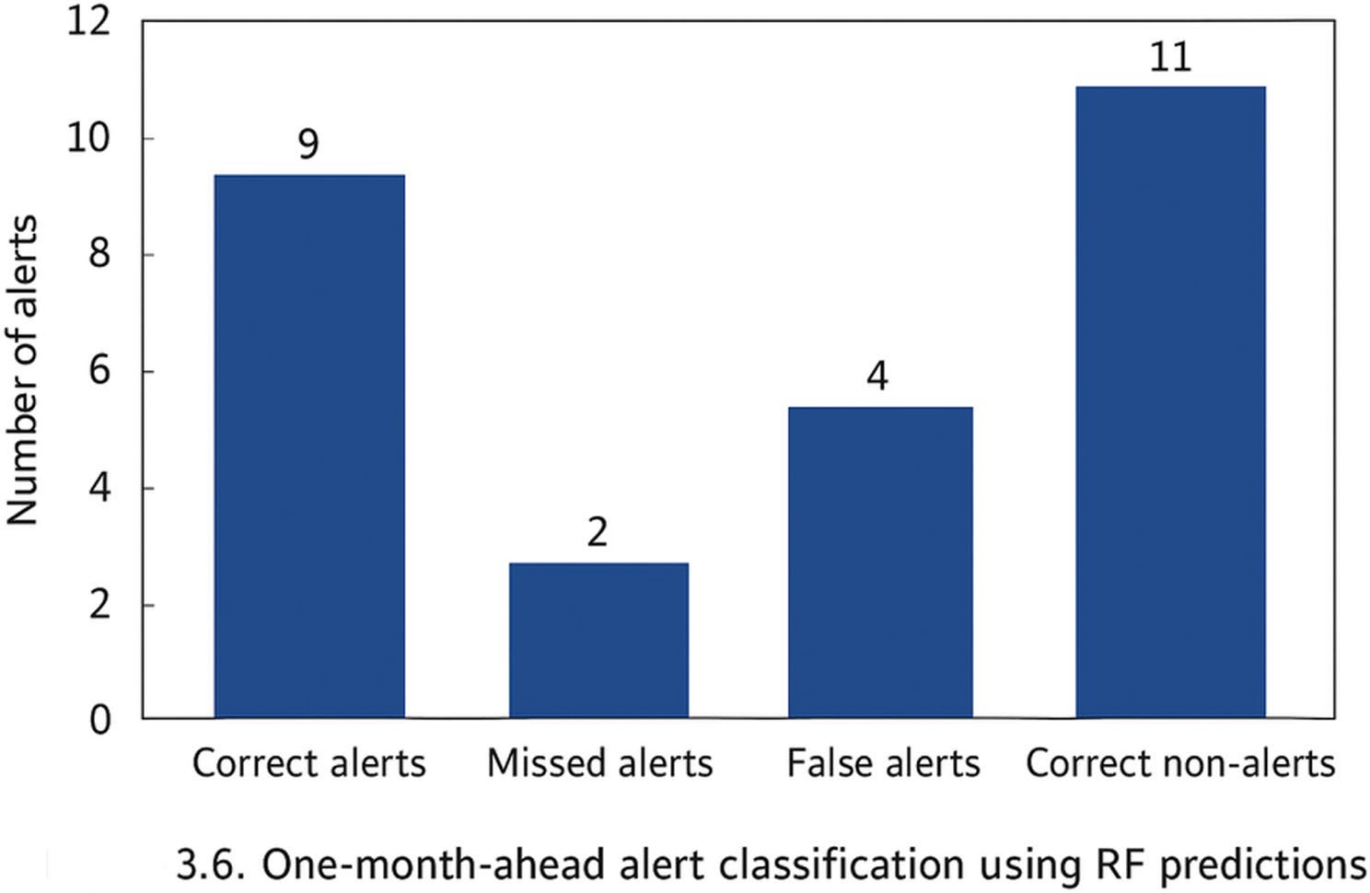
One-month-ahead alert classification using Random Forest predictions

The RF-based EWS achieved high sensitivity (0.82), indicating that most impending high-transmission periods were successfully flagged in advance, as illustrated by the one-month-ahead alert classifications shown in Fig. 6. The high negative predictive value (NPV; 0.86) suggests that months classified as low risk were generally associated with low observed case counts.

Figure 6 presents the distribution of prospective alert classifications across districts during the 2022 temporal test period. The results indicate generally stable alert behavior, with a balance between detecting high-risk months and limiting false alarms. While these findings suggest that the proposed EWS has the potential to support timely preparedness and targeted intervention planning, further evaluation through deployment studies or decision-focused simulations would be required to quantify its real-world operational impact and cost-effectiveness.

### External validation and generalizability

While the current model was trained and evaluated exclusively within Yogyakarta Province, its applicability to other ecological and demographic contexts remains to be empirically established. Formal external validation could not be conducted in this study due to data availability constraints; therefore, the findings should be interpreted as province-specific.

To support future assessments of generalizability, several validation pathways are outlined. First, cross-province testing is planned in provinces such as Central Java or East Java, which share broadly similar climatic conditions but differ in land-use patterns and urban structure, allowing evaluation of model stability under heterogeneous settings. Second, the Agile modeling framework permits iterative retraining as new data become available, enabling adaptation to varying rainfall regimes, population densities, and trajectories of urban expansion. Third, temporal generalization will be examined by withholding additional future years to assess robustness under interannual climate variability, including contrasting El Niño and La Niña conditions.

Taken together, these strategies outline a roadmap for future external validation rather than providing evidence of confirmed generalizability. Accordingly, the present results are best interpreted as context-specific to Yogyakarta Province, with broader applicability contingent upon subsequent multicenter and multi-period validation efforts.

## Discussion

This study demonstrates that an AI-based Random Forest ensemble can effectively model monthly dengue case counts in a hyperendemic Indonesian setting. The model integrates climatic, environmental, and socio-demographic predictors within an Agile system-development framework. The findings are consistent with previous studies highlighting the central role of temperature, rainfall, and humidity in shaping dengue transmission dynamics [24, 26, 27, 54–56], while extending existing work by incorporating multi-domain predictors within a unified and adaptive modeling architecture. By integrating environmental and socio-demographic variables, the model addresses key limitations of climate-only forecasting approaches [33, 37, 45, 48].

From a methodological perspective, the RF model exhibited strong predictive performance under temporal validation (R² = 0.86, RMSE = 5.72, MAE = 3.84), indicating its capacity to capture complex nonlinear relationships and interaction effects among heterogeneous predictors. While this study did not include a quantitative comparison with simple persistence-based baselines, the observed improvement over the GLM benchmark suggests that predictive gains are not driven by linear structure or distributional assumptions alone. These results are broadly consistent with previous applications of RF models for dengue prediction in Southeast Asia and Latin America [42, 44], supporting the suitability of ensemble learning approaches for modeling nonlinear epidemiological processes.

Model calibration and uncertainty assessment indicated that RF predictions were generally reliable across low to moderate transmission levels. However, mild under-coverage was observed, with a Prediction Interval Coverage Probability (PICP) of 0.91 relative to the nominal 0.95, alongside underestimation during outbreak peaks. Such behavior is common in overdispersed infectious disease time series, where extreme values are difficult to capture using ensemble averaging. Accordingly, while the model provides interpretable uncertainty estimates suitable for supporting near-term risk assessment, caution is warranted when interpreting predictions under extreme outbreak conditions.

The explainable feature-importance analysis provides insights with practical relevance for dengue control and preparedness. Rainfall lag-1, temperature, and relative humidity emerged as dominant climatic predictors, reinforcing evidence that weather-driven mosquito ecology strongly governs seasonal dengue dynamics. Environmental and socio-demographic variables—particularly built-up area and population density—also contributed meaningfully to model performance, highlighting the influence of urban structure and human exposure density on transmission intensity. These findings are consistent with prior studies emphasizing the interaction between urbanization, population pressure, and vector–human contact [24, 27], and underscore the value of integrating non-climatic predictors that are often omitted in meteorology-focused models [57, 58].

Beyond predictive accuracy, the proof-of-concept early-warning system (EWS) evaluation illustrates the potential operational relevance of the proposed framework. Using a one-month lead time and a district-specific 75th-percentile alert threshold, the RF-based EWS achieved high sensitivity (0.82) and a strong negative predictive value (0.86). These results indicate that the system is effective in identifying impending increases in transmission while reliably distinguishing low-risk periods, a desirable characteristic for prioritizing vector-control activities and minimizing unnecessary interventions. However, the EWS component is intended as an illustrative decision-support application rather than a fully deployed operational system.

In an operational setting, the proposed early-warning system could function as a monthly decision-support layer within existing district surveillance workflows. At the end of each reporting month, routinely collected case counts and updated climatic observations would be processed to generate a one-month-ahead district-level risk signal. Districts exceeding the predefined alert threshold could be prioritized for intensified larval source reduction, community mobilization, and preparedness planning, while low-risk districts would avoid unnecessary resource deployment. Importantly, the probabilistic nature of the forecasts allows public health officials to interpret alerts alongside uncertainty bounds, supporting risk-informed decision-making rather than binary responses.

From a systems-engineering perspective, embedding the predictive model within an Agile framework enhances adaptability by enabling iterative refinement as data patterns evolve. Rather than functioning as a static forecasting tool, the framework supports continuous updating of preprocessing rules, model parameters, and alert thresholds as new climatic, environmental, and epidemiological data become available. This design aligns with emerging digital health paradigms that emphasize flexibility, stakeholder feedback, and sustained performance in real-time surveillance systems [59, 60].

Several limitations should be acknowledged. First, environmental variables were derived from administratively aggregated land-use data, which may obscure fine-scale mosquito habitat heterogeneity. Future studies should consider higher-resolution remote-sensing indicators such as land-surface temperature or vegetation indices. Second, the presence of weak but statistically significant residual spatial autocorrelation (Moran’s I = 0.18, *p* = 0.041) suggests that adjacency-driven transmission processes were not fully captured, motivating future exploration of spatially explicit machine-learning extensions such as spatial RF or graph-based models. Third, although RF performed well overall, underestimation of extreme outbreak peaks indicates potential value in hybrid approaches that integrate ensemble learning with deep temporal architectures (e.g., CNN–LSTM).

Regarding external validity, the model was trained and evaluated exclusively using data from Yogyakarta Province, limiting direct generalization to other ecological or demographic contexts. Nevertheless, the Agile retraining workflow is inherently portable, allowing model adaptation under differing climatic regimes, urban configurations, and population structures. Future work will focus on cross-province validation, evaluation under contrasting climate variability (e.g., El Niño versus La Niña conditions), and incorporation of mobility or behavioral data to better capture human-driven transmission dynamics.

Overall, this study contributes to the growing field of AI-driven epidemiological modeling by demonstrating how ensemble machine learning can be integrated with an Agile system-development paradigm to produce a flexible, interpretable, and continuously updatable predictive framework. The results support a shift from static forecasting toward adaptive, data-driven decision-support systems capable of informing targeted dengue control and enhancing outbreak preparedness.

## Conclusion

This study provides evidence that an Agile–AI hybrid architecture can effectively model monthly dengue case-count patterns in a hyperendemic Indonesian setting. The framework combines a Random Forest ensemble with leakage-free preprocessing to ensure robust and unbiased prediction. By integrating climatic, environmental, and socio-demographic domains, the proposed framework addresses key limitations of traditional statistical approaches and offers a flexible structure capable of adapting to evolving epidemiological and climatic conditions.

The system demonstrated strong early-warning potential, characterized by high sensitivity and a robust negative predictive value, supporting its use as a decision-support tool for anticipating periods of elevated transmission and informing resource-efficient vector-control planning. While uncertainty estimates were generally interpretable across routine transmission levels, reduced reliability during outbreak peaks and the presence of residual spatial dependence highlight important considerations when interpreting extreme predictions and motivate further methodological refinement.

Overall, these findings position the proposed Agile–AI framework as a promising foundation for adaptive dengue surveillance and early-warning applications. With continued refinement through external validation, higher-resolution data integration, and spatially explicit extensions, the framework has strong potential for broader deployment across diverse ecological settings and for extension to other arboviral disease surveillance contexts.

## Supplementary Information

Below is the link to the electronic supplementary material.

Supplementary Material 1

## Data Availability

Climatic data were obtained from the Meteorology, Climatology, and Geophysics Agency (BMKG) of the Special Region of Yogyakarta. Environmental and socio-demographic data were sourced from Statistics Indonesia (Badan Pusat Statistik; BPS) and the Provincial Environmental Agency of the Special Region of Yogyakarta (Dinas Lingkungan Hidup Daerah Istimewa Yogyakarta). Dengue surveillance data were provided by the local health authority. Restrictions apply to the availability of these data, which were used under license for the current study and are therefore not publicly available. Data may be made available from the corresponding author upon reasonable request and with permission of the respective data-providing institutions.

## Abbreviations

AI: Artificial Intelligence
ARIMA: AutoRegressive Integrated Moving Average
BMKG: Meteorology, Climatology, and Geophysics Agency (Badan Meteorologi, Klimatologi, dan Geofisika)
BPS: Statistics Indonesia (Badan Pusat Statistik)
CNN: Convolutional Neural Network
DF: Dengue Fever
DENV: Dengue Virus
DHF: Dengue Hemorrhagic Fever
DIY: Daerah Istimewa Yogyakarta
EWS: Early-Warning System
GIS: Geographic Information System
GLM: Generalized Linear Model
INLA: Integrated Nested Laplace Approximation
IQR: Interquartile Range
LPDP: Endowment Fund for Education (Lembaga Pengelola Dana Pendidikan)
MAE: Mean Absolute Error
ML: Machine Learning
MPIW: Mean Prediction Interval Width
NB: Negative Binomial
NPV: Negative Predictive Value
PICP: Prediction Interval Coverage Probability
PPV: Positive Predictive Value
PRPB: Program Pendanaan Riset Pembangunan Berkelanjutan
R²: Coefficient of Determination
RF: Random Forest
RMSE: Root Mean Squared Error
SARIMA: Seasonal AutoRegressive Integrated Moving Average
VIF: Variance Inflation Factor
