# Supplementary material for: AI-based prediction of dengue incidence using climatic, environmental, and socio-demographic factors: an ensemble random forest approach with agile system development
Source: BMC Infect Dis. 2026 May 4;26:1199. doi: 10.1186/s12879-026-13270-1 (PMC13325788; doi:10.1186/s12879-026-13270-1)
Supplement: Supplementary file 1 — Supplementary Material 1 [file 12879_2026_13270_MOESM1_ESM.docx]

**Cover Letter**

To: The Editor-in-Chief

*BMC Infectious Diseases*

Dear Editor,

We are pleased to submit our manuscript entitled “AI-Based Prediction of Dengue Incidence using Climatic, Environmental, and Socio-Demographic Factors: An Ensemble Random Forest Approach with Agile System Development” for consideration for publication in *BMC Infectious Diseases*.

This study presents an AI-based predictive framework that integrates climatic, environmental, and socio-demographic factors to forecast dengue incidence in the Special Region of Yogyakarta, Indonesia. Utilizing an ensemble Random Forest model within an Agile System Development framework, the research introduces an adaptive, data-driven mechanism capable of continuous model retraining and recalibration as new climatic or epidemiological data become available.

The findings demonstrate that the proposed AI–Agile framework achieved superior predictive performance (R² = 0.86; RMSE = 5.72) compared to a Generalized Linear Model baseline (R² = 0.64). The model identifies rainfall (lag-1), relative humidity, and temperature as dominant climatic predictors, while built-up area and population density are key environmental and socio-demographic drivers of dengue transmission.

We believe this manuscript is well-suited for *BMC Infectious Diseases*, given the journal’s focus on infectious disease dynamics, epidemiological modeling, and innovative data analytics in public health. The integration of artificial intelligence and agile methodologies contributes a novel operational dimension to disease forecasting systems, aligning closely with the journal’s scope on digital epidemiology and data-driven surveillance.

This work has not been published elsewhere and is not under consideration by any other journal. All authors have read and approved the final manuscript and have no competing interests to declare. Ethical approval was obtained from the Ethics Board of the Faculty of Health Science, Respati University (Approval No. 0129.3/FIKES/PL/XI/2024).

We appreciate your consideration of our submission and look forward to the opportunity to contribute to *BMC Infectious Diseases*.

Sincerely,

**Nur Alvira, MPH**

Tel: [+628176616928]

## ****Manuscript Title:****

## **AI-Based Prediction of Dengue Incidence using Climatic, Environmental, and Socio-Demographic Factors: An Ensemble Random Forest Approach with Agile System Development**

## ****Author Information****

## **Corresponding Author:**

## **Nur Alvira, MPH**

## Doctoral Candidate, Faculty of Public Health, *Doctoral Program, Public Health Study Program*, Universitas Airlangga

Mulyorejo, Kec. Mulyorejo, Surabaya, Jawa Timur 60115
📧 Email: [[alvirapascawati@gmail.com](mailto:alvirapascawati@gmail.com) ; [nur.alvira.pascawati-2024@fkm.unair.ac.id](mailto:nur.alvira.pascawati-2024@fkm.unair.ac.id)]

📞 Phone: [+628176616928]
🔗 ORCID: [https://orcid.org/0000-0003-2804-7620]

**Co-Authors:**

1. **Tri Baskoro Tunggul Satoto, M.Sc., Ph.D.**

Department of Parasitology, Faculty of Medicine, Public Health, and Nursing, Universitas Gadjah Mada, Yogyakarta, Indonesia

ORCID: [https://orcid.org/0000-0003-4443-4751]

2. **Nur Alvira Pascawati**

Faculty of Public Health, Doctoral Program, Public Health Study Program, Universitas Airlangga, Surabaya, Indonesia

ORCID: [https://orcid.org/0000-0003-2804-7620]

3. **Roger Frutos**

CIRAD, University of Montpellier, Montpellier, France

ORCID: [https://orcid.org/[0000-0002-8926-3119](https://orcid.org/0000-0002-8926-3119)]

4. **Erizal**

Faculty of Science Technology, Information System Study Program, Universitas Respati, Yogyakarta, Indonesia

5. **Triwibowo Ambar Garjito**

The vector-borne and zoonotic diseases research group at National Research and Innovation Agency of Indonesia

ORCID: [https://orcid.org/0000-0002-7697-9759]

## ****Author Contributions (CRediT Statement)****

- **Conceptualization:** Nur Alvira Pascawati, Tri Baskoro Tunggul Satoto, Roger Frutos
- **Data Curation:** Nur Alvira Pascawati, Erizal
- **Methodology:** Nur Alvira Pascawati, Roger Frutos, Erizal
- **Formal Analysis:** Nur Alvira Pascawati
- **Validation:** Roger Frutos, Triwibowo Ambar Garjito
- **Writing Original Draft:** Nur Alvira Pascawati
- **Writing Review & Editing:** Nur Alvira Pascawati, Tri Baskoro Tunggul Satoto
- **Supervision:** Tri Baskoro Tunggul Satoto, Roger Frutos

All authors have read and approved the final version of this manuscript.

## ****Funding Statement****

## This research was supported by the **Sustainable Development Research Grant (Program Pendanaan Riset Pembangunan Berkelanjutan – PRPB)** under the International Collaboration Scheme, administered by the **Directorate General of Higher Education, Research, and Technology**, **Ministry of Higher Education, Science, and Technology of the Republic of Indonesia**, in collaboration with **Universitas Gadjah Mada** (Contract No. 014/E5/PG.02.00/PRPB BATCH 2/2024). Additional financial support was provided through the **Center for Higher Education Funding and Assessment (PPAPT)** and the **Endowment Fund for Education (LPDP)**, **Ministry of Finance of the Republic of Indonesia**, via the **Indonesian Education Scholarship** scheme.

## ****Ethical Approval****

Ethical approval for this study was obtained from the **Faculty of Health Science Ethics Board, Respati University**, Yogyakarta, Indonesia (Approval No. 0129.3/FIKES/PL/XI/2024).

## ****Data Availability Statement****

## All datasets generated and analyzed during this study are available from the corresponding author upon reasonable request. Climatic, environmental, and socio-demographic data were obtained from:

- Meteorology, Climatology, and Geophysics Agency (BMKG) of Yogyakarta
- Statistics Indonesia (BPS)
- Yogyakarta Provincial Health Office

## ****Competing Interests****

The authors declare **no competing interests**

## ****Acknowledgments****

The authors would like to express their sincere appreciation to **Universitas Gadjah Mada (UGM)** for institutional support throughout this study. Special acknowledgment is extended to the **Meteorology, Climatology, and Geophysics Agency (BMKG) of Yogyakarta**, the **Yogyakarta Provincial Health Office**, the **Regional Spatial and Environmental Agency**, and **Statistics Indonesia (BPS)** for providing access to the climatic, environmental, and socio-demographic datasets essential for this research
